# Supplementary material for: Bio-Guided Fractionation of Prenylated Benzaldehyde Derivatives as Potent Antimicrobial and Antibiofilm from Ammi majus L. Fruits-Associated Aspergillus amstelodami
Source: Molecules. 2019 Nov 14;24(22):4118. doi: 10.3390/molecules24224118 (PMC6891696; doi:10.3390/molecules24224118)
Supplement: Supplementary file 1 [file molecules-24-04118-s001.zip › S6 fileTables of MBICs values for antibiofilm activities against different microorganism strains..pdf]

Table S5 A: Antibiofilm activity of the plant total extract:

Sample conc. (µg/ml)	*Pseudomonas aeruginosa*	*staphylococcus aureus*	*Streptococcus mutans*	*Escherichia coli*	*Candida albicans*
1000	73.25±1.5	100.00±0.00	0±0.00	100.00±0.00	0±0.00
500	43.12±1.2	100.00±0.00	0±0.00	100.00±0.00	0±0.00
250	26.17±1.5	91.25±1.5	0±0.00	100.00±0.00	0±0.00
125	11.35±2.1	74.16±1.2	0±0.00	90.35±1.3	0±0.00
62.5	5.12±0.63	53.21±0.63	0±0.00	76.32±0.58	0±0.00
31.25	0±0.00	29.24±1.5	0±0.00	49.31±0.72	0±0.00
15.63	0±0.00	15.32±0.63	0±0.00	23.24±2.1	0±0.00
7.81	0±0.00	6.14±1.3	0±0.00	8.31±1.5	0±0.00
0	0±0.0	0±0.0	0±0.00	0±0.0	0±0.00
MBIC	>1000	500	-	250	-

Table S5 B: Antibiofilm activity of the fungal extract:

Sample conc. (µg/ml)	*Pseudomonas aeruginosa*	*staphylococcus aureus*	*Streptococcus mutans*	*Escherichia coli*	*Candida albicans*
**1000**	**100±0.0**	**100±0.00**	**100±0.00**	**100±0.0**	**100±0.0**
**500**	**100±0.0**	**100±0.00**	**100±0.00**	**100±0.0**	**100±0.0**
**250**	**100±0.0**	**100±0.00**	**100±0.00**	**100±0.0**	**100±0.0**
**125**	**89.34±1.3**	**100±0.00**	**82.31±0.58**	**100±0.0**	**79.35±0.72**
**62.5**	**61.25±2.1**	**100±0.00**	**57.34±0.63**	**82.16±0.92**	**52.14±2.1**
**31.25**	**37.82±0.72**	**83.21±0.72**	**31.34±2.1**	**59.32±1.5**	**28.14±2.1**
**15.63**	**19.86±2.5**	**74.25±0.92**	**14.34±1.5**	**32.14±1.3**	**14.32±1.3**
**7.81**	**0±0.0**	**42.16±1.5**	**0±0.0**	**11.35±0.63**	**5.37±0.58**
**0**	**0±0.0**	**0.00±0.00**	**0±0.0**	**0±0.0**	**0±0.0**
**MBIC**	**250**	**62.5**	**250**	**125**	**250**

Table S5 C: Antibiofilm activity of the fungal fraction I:

Sample conc. (µg/ml)	*Pseudomonas aeruginosa*	*staphylococcus aureus*	*Streptococcus mutans*	*Escherichia coli*	*Candida albicans*
**1000**	**100±0.0**	**100±0.0**	**100±0.0**	**100±0.0**	**100±0.0**
**500**	**100±0.0**	**100±0.0**	**100±0.0**	**100±0.0**	**100±0.0**
**250**	**81.32±2.1**	**100±0.0**	**100±0.0**	**100±0.0**	**100±0.0**
**125**	**48.35±1.3**	**100±0.0**	**86.32±2.1**	**100±0.0**	**81.34±1.5**
**62.5**	**26.37±0.58**	**78.15±2.1**	**57.32±1.5**	**100±0.0**	**63.25±2.1**
**31.25**	**12.35±1.5**	**39.35±0.63**	**33.46±0.58**	**91.25±1.3**	**37.35±0.58**
**15.63**	**6.12±1.3**	**22.63±1.2**	**26.35±0.63**	**66.34±1.5**	**17.31±2.1**
**7.81**	**0±0.00**	**17.32±0.58**	**10.32±0.63**	**49.32±1.2**	**4.32±1.5**
**0**	**0±0.0**	**5.32±1.5**	**6.31±1.5**	**26.35±1.5**	**0±0.0**
**MBIC**	**500**	**125**	**250**	**62.5**	**500**

Table S5 D: Antibiofilm activity of the fungal fraction II :

Sample conc. (μg/ml)	*Pseudomonas aeruginosa*	*staphylococcus aureus*	*Streptococcus mutans*	*Escherichia coli*	*Candida albicans*
**1000**	**100±0.0**	**100±0.0**	**100±0.0**	**100±0.0**	**100±0.0**
**500**	**100±0.0**	**100±0.0**	**100±0.0**	**100±0.0**	**100±0.0**
**250**	**100±0.0**	**100±0.0**	**100±0.0**	**100±0.0**	**100±0.0**
**125**	**83.25±1.2**	**92.35±0.63**	**100±0.0**	**100±0.0**	**71.35±1.2**
**62.5**	**64.35±2.1**	**76.34±1.3**	**71.32±1.4**	**100±0.0**	**53.25±0.63**
**31.25**	**49.35±0.92**	**60.14±2.1**	**52.14±0.72**	**81.35±2.3**	**36.25±2.5**
**15.63**	**24.32±1.5**	**42.15±1.5**	**31.25±1.5**	**56.32±2.1**	**14.36±1.2**
**7.81**	**9.35±0.63**	**24.13±0.92**	**14.32±1.2**	**36.25±1.5**	**6.24±3.1**
**0**	**0±0.0**	**12.35±2.1**	**8.32±0.63**	**22.74±0.63**	**0±0.0**
**MBIC**	**250**	**250**	**125**	**62.5**	**250**

Table S5 E: Antibiofilm activity of the fungal fraction III:

**Sample conc. (µg/ml)**	***Pseudomonas aeruginosa***	***staphylococcus aureus***	***Streptococcus mutans***	***Escherichia coli***	***Candida albicans***
**1000**	**100±0.0**	**100±0.0**	**100±0.0**	**100±0.0**	**100±0.0**
**500**	**100±0.0**	**100±0.0**	**100±0.0**	**100±0.0**	**100±0.0**
**250**	**100±0.0**	**100±0.0**	**100±0.0**	**100±0.0**	**100±0.0**
**125**	**100±0.0**	**100±0.0**	**100±0.0**	**100±0.0**	**100±0.0**
**62.5**	**83.25±1.5**	**100±0.0**	**100±0.0**	**100±0.0**	**100±0.0**
**31.25**	**64.12±0.63**	**100±0.0**	**100±0.0**	**100±0.0**	**88.32±1.2**
**15.63**	**46.34±0.58**	**83.25±1.3**	**76.35±0.73**	**100±0.0**	**70.34±2.1**
**7.81**	**19.25±1.2**	**57.24±2.1**	**49.86±1.5**	**92.15±1.6**	**52.19±1.5**
**0**	**0±0.0**	**29.12±0.92**	**21.35±2.1**	**78.25±1.3**	**38.15±2.2**
MBIC	125	31.25	31.25	15.63	62.5

Table S5 F: Antibiofilm activity of the Dihydroauroglaucin:

**Sample conc. (µg/ml)**	***Pseudomonas aeruginosa***	***staphylococcus aureus***	***Streptococcus mutans***	***Escherichia coli***	***Candida albicans***
**125**	**100± 0.00**	**100± 0.00**	**100± 0.00**	**100± 0.00**	**100± 0.00**
**62.5**	**100± 0.00**	**100± 0.00**	**100± 0.00**	**100± 0.00**	**100± 0.00**
**31.25**	**100± 0.00**	**100± 0.00**	**100± 0.00**	**100± 0.00**	**100± 0.00**
**15.63**	**81.35± 1.6**	**100± 0.00**	**100± 0.00**	**100± 0.00**	**100± 0.00**
**7.81**	**57.32± 2.1**	**100± 0.00**	**90.38± 1.6**	**100± 0.00**	**86.76± 0.58**
**3.9**	**22.36± 0.63**	**93.25± 2.1**	**72.14 ± 0.63**	**71.35± 2.1**	**65.38 ± 2.1**
**1.95**	**10.63 ± 1.2**	**16.34 ± 1.8**	**54.32± 1.5**	**60.28 ± 1.5**	**33.20± 0.63**
**0**	**0± 0.00**	**0± 0.00**	**0± 0.00**	**0± 0.00**	**0± 0.00**
**MBIC**	**31.25**	**7.81**	**15.63**	**7.81**	**15.63**

S6 file: Tables of MBICs values for antibiofilm activities against different microorganism strains.
