# Supplementary material for: Bio-Guided Fractionation of Prenylated Benzaldehyde Derivatives as Potent Antimicrobial and Antibiofilm from Ammi majus L. Fruits-Associated Aspergillus amstelodami
Source: Molecules. 2019 Nov 14;24(22):4118. doi: 10.3390/molecules24224118 (PMC6891696; doi:10.3390/molecules24224118)
Supplement: Supplementary file 1 [file molecules-24-04118-s001.zip › S1 file BLAST Alignment for identification of A. amestoldami.pdf]

Description	Max score	Total score	Query cover	E value	Ident	Accession
Aspergillus amstelodami isolate FBKL3.0174 internal transcribed spacer 1, partial sequence; 5.8S ribosomal RNA gene and internal transcribed spacer 2, co	852	852	98%	0.0	99%	KY828904.1
Aspergillus amstelodami genomic DNA containing ITS1, 5.8S rRNA gene and ITS2, isolate TUHT55	852	852	99%	0.0	99%	LN482451.1
Aspergillus chevalieri strain 1963 18S ribosomal RNA gene, partial sequence; internal transcribed spacer 1, 5.8S ribosomal RNA gene, and internal transcrib	850	850	99%	0.0	99%	KX696384.1
Aspergillus chevalieri genomic DNA containing ITS1, 5.8S rRNA gene and ITS2, isolate TUHT82	850	850	99%	0.0	99%	LN482478.1
Aspergillus cristatus isolate 97 internal transcribed spacer 1, partial sequence; 5.8S ribosomal RNA gene and internal transcribed spacer 2, complete sequen	848	848	98%	0.0	99%	MH345937.1
Aspergillus amstelodami isolate BAB-6506 small subunit ribosomal RNA gene, partial sequence; internal transcribed spacer 1, 5.8S ribosomal RNA gene, an	848	848	98%	0.0	99%	MF319924.1
Aspergillus intermedius strain E20 18S ribosomal RNA gene, partial sequence; internal transcribed spacer 1, 5.8S ribosomal RNA gene, and internal transcri	848	848	98%	0.0	99%	KX696385.1
Aspergillus chevalieri strain E9 18S ribosomal RNA gene, partial sequence; internal transcribed spacer 1, 5.8S ribosomal RNA gene, and internal transcribed	848	848	98%	0.0	99%	KX696378.1
Aspergillus pseudoglaucus internal transcribed spacer 1, partial sequence; 5.8S ribosomal RNA gene and internal transcribed spacer 2, complete sequence;	848	848	98%	0.0	99%	KX858814.1
Aspergillus ruber genomic DNA containing ITS1, 5.8S rRNA gene and ITS2, isolate TUHT74	848	848	98%	0.0	99%	LN482470.1
Aspergillus cristatus strain gxlbc3 internal transcribed spacer 1, partial sequence; 5.8S ribosomal RNA gene and internal transcribed spacer 2, complete sequ	848	848	98%	0.0	99%	KJ018706.1
Eurotium cristatum strain EN220 18S ribosomal RNA gene, partial sequence; internal transcribed spacer 1, 5.8S ribosomal RNA gene, and internal transcribe	848	848	98%	0.0	99%	JQ743649.1
Aspergillus montevidensis genomic DNA sequence contains ITS1, 5.8S rRNA gene, ITS2, isolate UTHSCSA:DI15-22	846	846	98%	0.0	99%	LT627260.1
Eurotium amstelodami strain SGE21 18S ribosomal RNA gene, partial sequence; internal transcribed spacer 1, 5.8S ribosomal RNA gene, and internal trans	846	846	98%	0.0	99%	JX232268.1
Aspergillus sp. strain ALE-121 internal transcribed spacer 1, partial sequence; 5.8S ribosomal RNA gene and internal transcribed spacer 2, complete sequen	845	845	99%	0.0	99%	MF380814.1
Aspergillus chevalieri isolate 1100 internal transcribed spacer 1, partial sequence; 5.8S ribosomal RNA gene and internal transcribed spacer 2, complete seq	845	845	98%	0.0	99%	KT826616.1
Aspergillus chevalieri isolate BAB-6505 internal transcribed spacer 1, partial sequence; 5.8S ribosomal RNA gene and internal transcribed spacer 2, complet	845	845	99%	0.0	99%	MF319917.1
Aspergillus amstelodami genomic DNA containing ITS1, 5.8S rRNA gene and ITS2, strain TUHT140	845	845	99%	0.0	99%	LN482536.1
Eurotium rubrum strain Mehrabi65 16S ribosomal RNA gene, partial sequence	845	845	98%	0.0	99%	JN032757.1
Aspergillus cristatus isolate E6 18S ribosomal RNA gene, partial sequence; internal transcribed spacer 1, 5.8S ribosomal RNA gene, and internal transcribed	843	843	99%	0.0	99%	MG199954.1
Aspergillus chevalieri isolate E3 18S ribosomal RNA gene, partial sequence; internal transcribed spacer 1, 5.8S ribosomal RNA gene, and internal transcribe	843	843	99%	0.0	99%	MG199953.1
Aspergillus chevalieri isolate E2 18S ribosomal RNA gene, partial sequence; internal transcribed spacer 1, 5.8S ribosomal RNA gene, and internal transcribe	843	843	99%	0.0	99%	MG199952.1

```
Query  1    GCGTGGCCACGGCCCGCCGGAGACTAACATTTGAACGCTGTCTGAAGTTTGCAGTCTGAG  60
            ||||||||||||||||||||||||||||||||||||||||||||||||||||||||||||
Sbjct  78   GCGTGGCCACGGCCCGCCGGAGACTAACATTTGAACGCTGTCTGAAGTTTGCAGTCTGAG  137

Query  61   TTTTTAGTTAAACAATCGTTAAAACTTTCAACAACGGATCTCTTGGTTCCGGCATCGATG  120
            ||||||||||||||||||||||||||||||||||||||||||||||||||||||||||||
Sbjct  138  TTTTTAGTTAAACAATCGTTAAAACTTTCAACAACGGATCTCTTGGTTCCGGCATCGATG  197

Query  121  AAGAACGCAGCGAAATGCGATAATTAATGTGAATTGCAGAATTCAGTGAATCATCGAGTC  180
            ||||||||||||||||||||||||||||||||||||||||||||||||||||||||||||
Sbjct  198  AAGAACGCAGCGAAATGCGATAATTAATGTGAATTGCAGAATTCAGTGAATCATCGAGTC  257

Query  181  TTTGAACGCACATTGCGCCCCCTGGTATTCCGGGGGGCATGCCTGTCCGAGCGTCATTGC  240
            ||||||||||||||||||||||||||||||||||||||||||||||||||||||||||||
Sbjct  258  TTTGAACGCACATTGCGCCCCCTGGTATTCCGGGGGGCATGCCTGTCCGAGCGTCATTGC  317

Query  241  TGCCCTCAAGCACGGCTTGTGTGTTGGGCTTCCGTCCCTGGCAACGGGGACGGGCCCAAA  300
            ||||||||||||||||||||||||||||||||||||||||||||||||||||||||||||
Sbjct  318  TGCCCTCAAGCACGGCTTGTGTGTTGGGCTTCCGTCCCTGGCAACGGGGACGGGCCCAAA  377

Query  301  AGGCAGTGGCGGCACCATGTCTGGTCCTCGAGCGTATGGGGCTTTGTCACCCGCTCCCGT  360
            ||||||||||||||||||||||||||||||||||||||||||||||||||||||||||||
```

```
Sbjct  378  AGGCAGTGGCGGCACCATGTCTGGTCCTCGAGCGTATGGGGCTTTGTCACCCGCTCCCGT  437


Query  361  AGGTCCAGCTGGCAGCTAGCCTCGCAACCAATCTTTTTAACCAGGTTGACCTCGGATCAG  420
            ||||||||||||||||||||||||||||||||||||||||||||||||||||||||||||
Sbjct  438  AGGTCCAGCTGGCAGCTAGCCTCGCAACCAATCTTTTTAACCAGGTTGACCTCGGATCAG  497


Query  421  GTAGGGATACCCGCTGAACTTAAGCATATCAAAAAAACCGGAGGAA  465
            ||||||||||||||||||||||||||||||||||  |||||||||
Sbjct  498  GTAGGGATACCCGCTGAACTTAAGCATATCAAAANNCCGGAGGAA  542
```

S1 file: BLAST Alignment for identification of *A. amestoldami*
