# Supplementary material for: Bio-Guided Fractionation of Prenylated Benzaldehyde Derivatives as Potent Antimicrobial and Antibiofilm from Ammi majus L. Fruits-Associated Aspergillus amstelodami
Source: Molecules. 2019 Nov 14;24(22):4118. doi: 10.3390/molecules24224118 (PMC6891696; doi:10.3390/molecules24224118)
Supplement: Supplementary file 1 [file molecules-24-04118-s001.zip › S2 file NMR Charts of isolated Compounds.pdf]

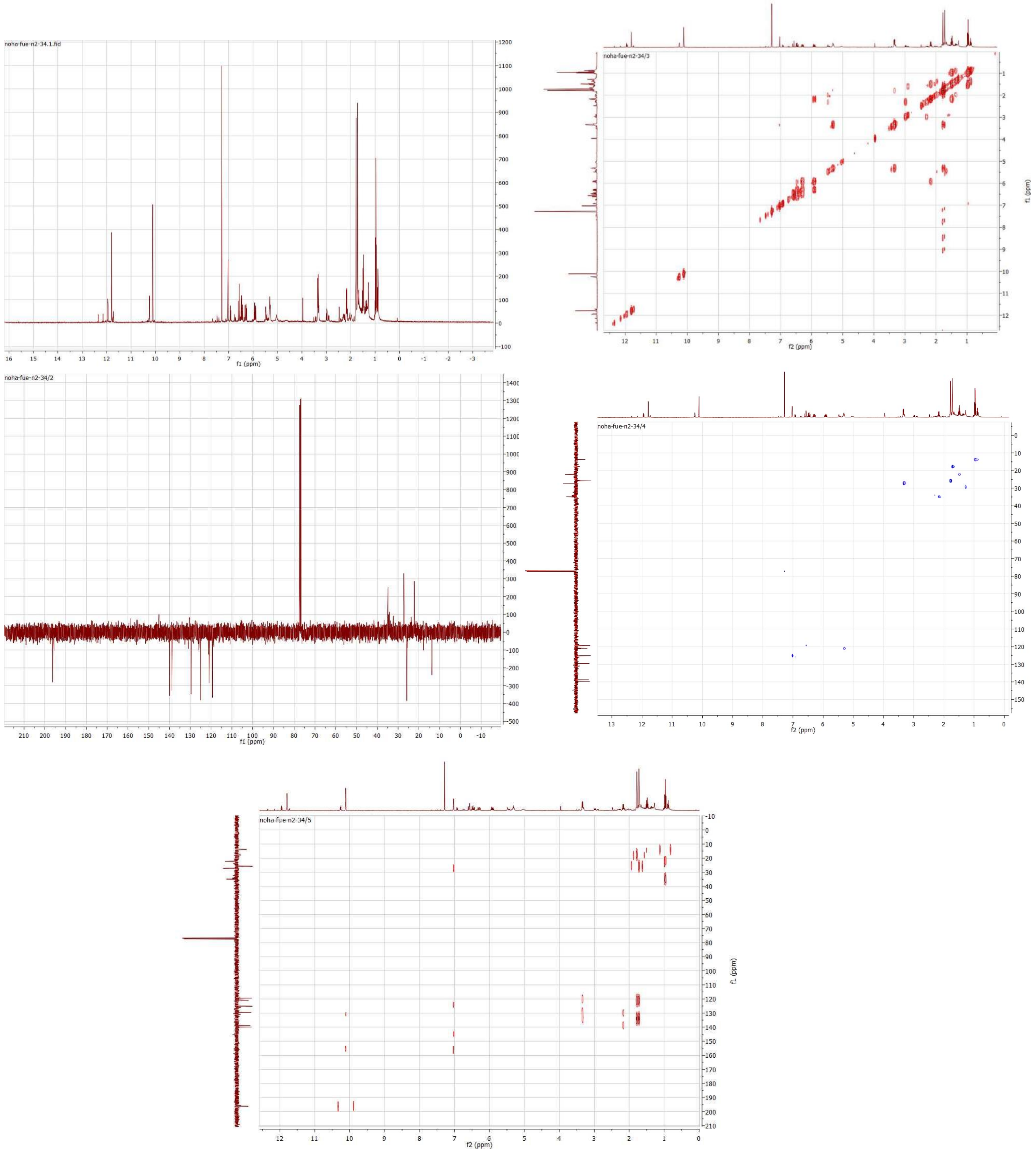

Fig S2 A: Spectral data of Compound 1 (Dihydroauroglaucin)

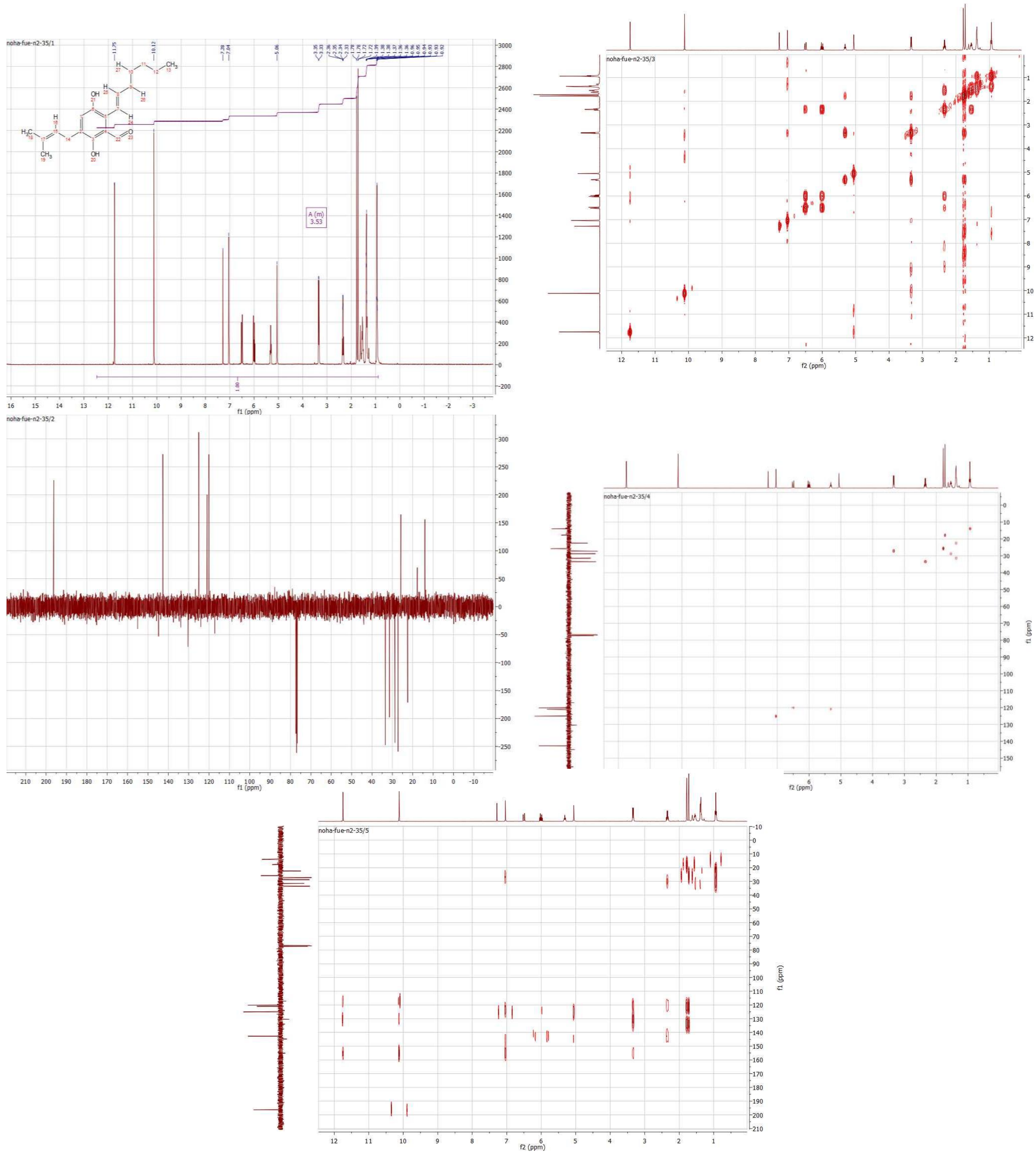

Fig S2 B: Spectral data of Compound 2 (Tetrahydroauroglaucin)

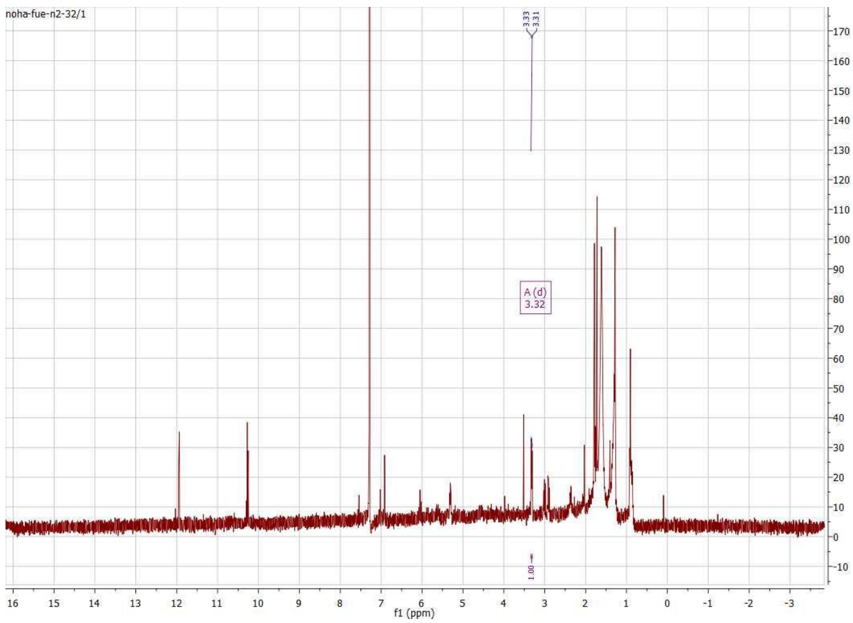

Fig S2 C: Spectral data of Compound 3 (2-(-3,6-dihydroxyhepta-1,4-dien-1-yl)-3,6-dihydroxy-5-(dimethylallyl) benzaldehyde)

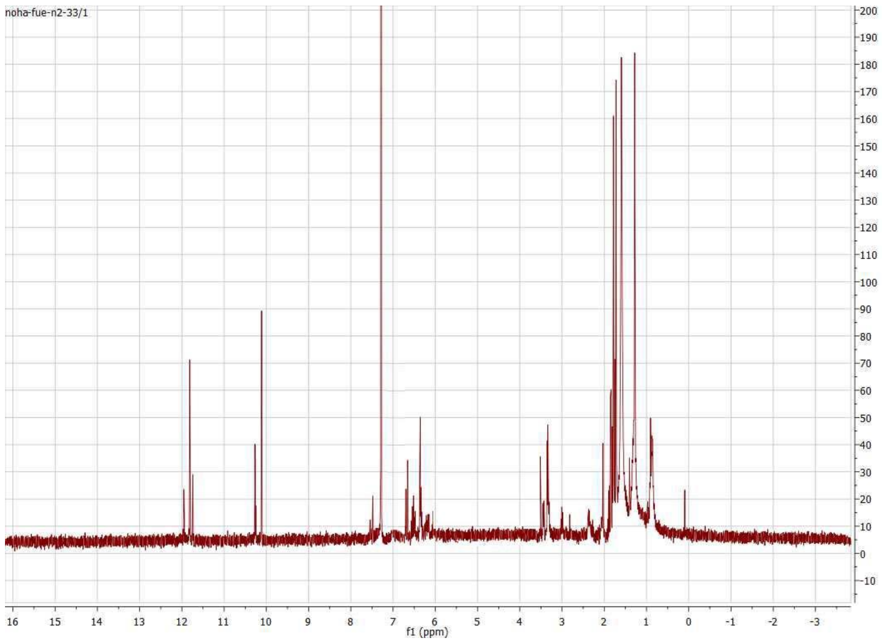

Fig S2 D: Spectral data of Compound 4 (Isotetrahydroauroglaucin)

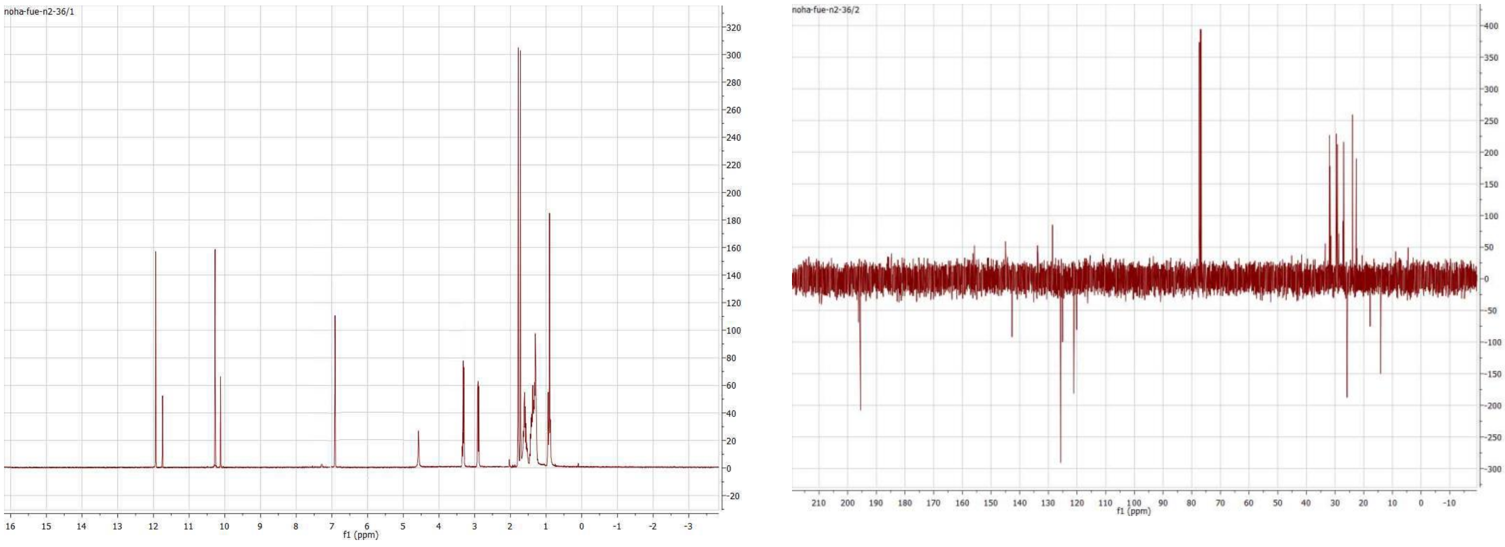

Fig S2 E: Spectral data of Compound 5 (Flavoglaucin)

S2 file: NMR Charts of isolated Compounds
