# Supplementary material for: Bio-Guided Fractionation of Prenylated Benzaldehyde Derivatives as Potent Antimicrobial and Antibiofilm from Ammi majus L. Fruits-Associated Aspergillus amstelodami
Source: Molecules. 2019 Nov 14;24(22):4118. doi: 10.3390/molecules24224118 (PMC6891696; doi:10.3390/molecules24224118)
Supplement: Supplementary file 1 [file molecules-24-04118-s001.zip › S3 file Figures for Antimicrobial activities using microdilution assay.pdf]

I- Antimicrobial activity using microdilution assay

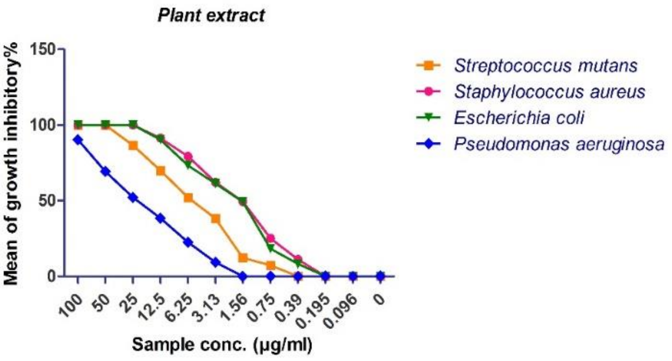

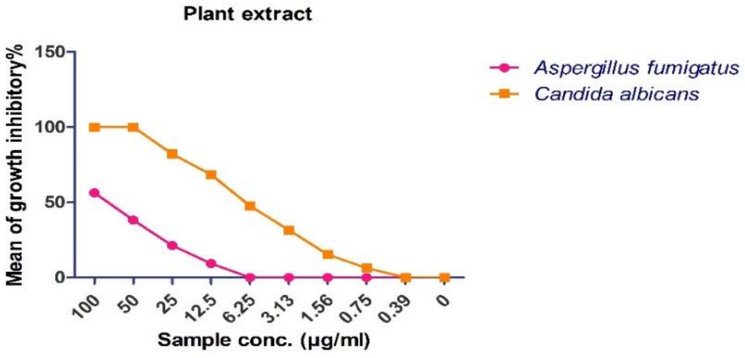

Antibacterial activity of the plant extract **Antifungal activity of the plant extract**

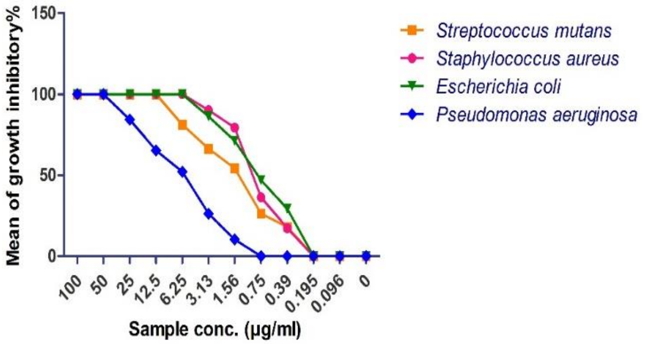

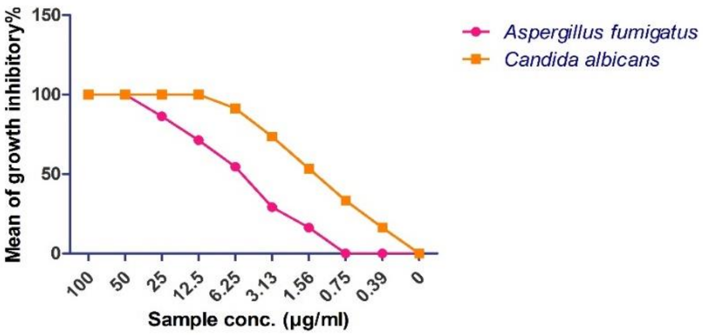

Antibacterial activity of the Fungal extract **Antifungal activity of the fungal extract**

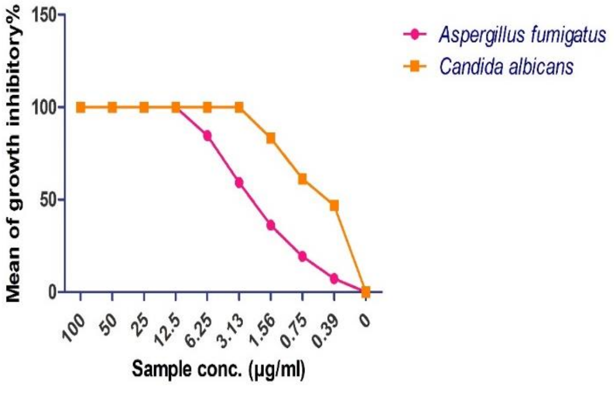

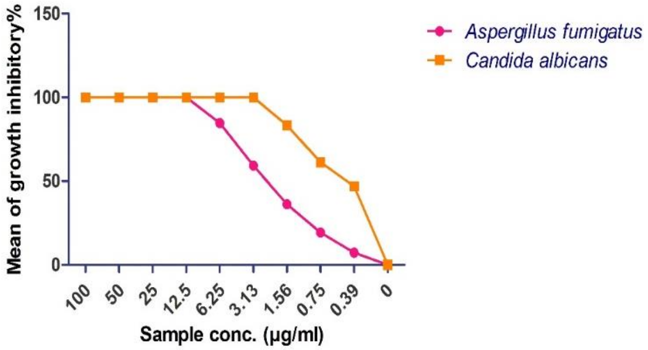

Ciprofloxacin effect on different bacterial strains **Amphotericin B effect on different fungal strains**

N.B *Aspergillus fumigatus* results were very low, so it was neglected during the further studies

Antimicrobial activity using microdilution assay (continue)

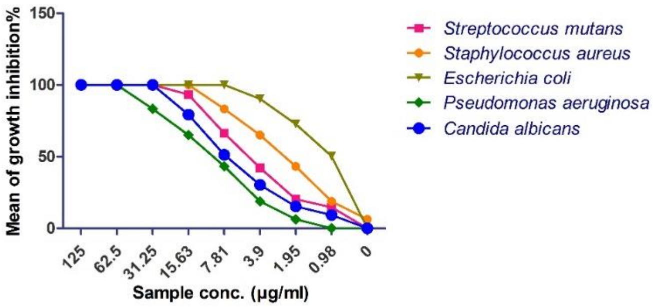

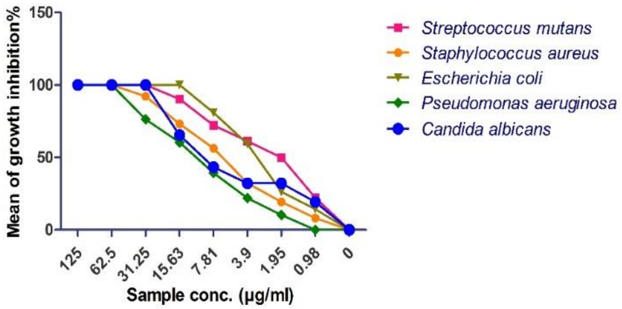

Antimicrobial activity of fraction 1

Antimicrobial activity of fraction 2

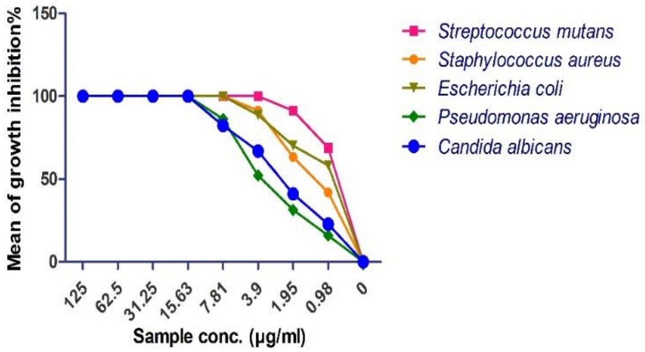

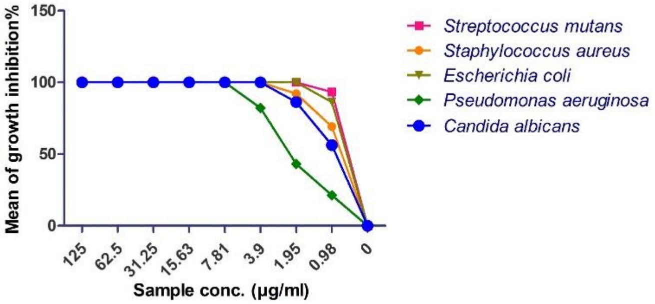

Antimicrobial activity of fraction 3

Antimicrobial activity of Dihydroauroglauc
