# Supplementary material for: Bio-Guided Fractionation of Prenylated Benzaldehyde Derivatives as Potent Antimicrobial and Antibiofilm from Ammi majus L. Fruits-Associated Aspergillus amstelodami
Source: Molecules. 2019 Nov 14;24(22):4118. doi: 10.3390/molecules24224118 (PMC6891696; doi:10.3390/molecules24224118)
Supplement: Supplementary file 1 [file molecules-24-04118-s001.zip › S4 fileTables of MICs values for antimicrobial activities using microdilution assay.pdf]

Table S4 A: Antimicrobial activity of Total plant Extract against *Stapylococcus aureus*

Sample conc. (μg/ml)	Mean of growth inhibitory%	S.D.
**100**	**100.00**	**0.00**
**50**	**100.00**	**0.00**
**25**	**100.00**	**0.00**
**12.5**	**91.25**	**2.10**
**6.25**	**79.35**	**1.20**
**3.13**	**62.14**	**0.77**
**1.56**	**49.38**	**1.02**
**0.75**	**25.13**	**1.30**
**0.39**	**11.20**	**0.00**
**0**	**0.00**	**0.00**
**MIC90 (μg/ml)**	**10.71**	
**MIC(μg/ml)**	**25**	

Table S4 B: Antimicrobial activity of Plant Extract against *Streptococcus mutans*

Sample conc. (μg/ml)	Mean of growth inhibitory%	S.D.
**100**	**100.00**	**0.00**
**50**	**100.00**	**0.00**
**25**	**86.50**	**1.20**
**12.5**	**69.88**	**0.58**
**6.25**	**52.14**	**0.72**
**3.13**	**38.16**	**1.50**
**1.56**	**12.34**	**2.10**
**0.75**	**7.32**	**0.72**
**0.39**	**0.00**	**0.00**
**0**	**0.00**	**0.00**
**MIC90 (μg/ml)**	**31.48**	
**MIC(μg/ml)**	**50**	

Table S4 C: Antimicrobial activity of Plant Extract against *Escherichia coli*

Sample conc. (µg/ml)	Mean of growth inhibitory%	S.D.
**100**	**100.00**	**0.00**
**50**	**100.00**	**0.00**
**25**	**100.00**	**0.00**
**12.5**	**90.31**	**0.63**
**6.25**	**73.25**	**1.20**
**3.13**	**61.37**	**0.58**
**1.56**	**49.32**	**2.10**
**0.75**	**18.35**	**1.30**
**0.39**	**8.31**	**1.20**
**0**	**0.00**	**0.00**
**MIC90 (µg/ml)**	**12.1**	
**MIC(µg/ml)**	**25**	

Table S4 D: Antimicrobial activity of Plant Extract against *Pseudomonas aeruginosa*

Sample conc. (µg/ml)	Mean of growth inhibitory%	S.D.
**100**	**90.35**	**2.10**
**50**	**69.34**	**1.60**
**25**	**52.17**	**0.72**
**12.5**	**38.38**	**1.50**
**6.25**	**22.60**	**1.30**
**3.13**	**9.35**	**0.58**
**1.56**	**0.00**	**0.00**
**0.75**	**0.00**	**0.00**
**0.39**	**0.00**	**0.00**
**0**	**0.00**	**0.00**
**MIC90 (µg/ml)**	**99.16**	
**MIC(µg/ml)**	**>100**	

Table S4 E: Antimicrobial activity of Plant Extract against
Candida albicans

Sample conc. (µg/ml)	Mean of growth inhibitory%	S.D.
100	100.00	0.00
50	100.00	0.00
25	82.14	0.63
12.5	68.42	1.50
6.25	47.69	2.10
3.13	31.52	0.63
1.56	15.37	2.1
0.75	6.31	0.58
0.39	0.00	0.00
0	0.00	0.00
MIC90 (µg/ml)	36.0	
MIC(µg/ml)	50	

Table S4 F: Antimicrobial activity of Plant Extract against
Aspergillus fumigatus

Sample conc. (µg/ml)	Mean of growth inhibitory%	S.D.
100	56.31	1.50
50	38.21	0.58
25	21.37	2.10
12.5	9.35	1.30
6.25	0.00	0.00
3.13	0.00	0.00
1.56	0.00	0.00
0.75	0.00	0.00
0.39	0.00	0.00
0	0.00	0.00
MIC90 (µg/ml)	>100	
MIC(µg/ml)	>100	

Table S4 g: Antimicrobial activity of fungus Extract against
Staphylococcus aureus

Sample conc. (μg/ml)	Mean of growth inhibitory%	S.D.
100	100.00	0.00
50	100.00	0.00
25	100.00	0.00
12.5	100.00	0.00
6.25	100.00	0.00
3.13	90.14	0.58
1.56	79.49	2.1
0.75	36.38	0.72
0.39	17.32	1.30
0	0.00	0.00
MIC90 (μg/ml)	3.08	
MIC(μg/ml)	6.25	

Table S4 H: Antimicrobial activity of fungus Extract against
Streptococcus mutans

Sample conc. (μg/ml)	Mean of growth inhibitory%	S.D.
100	100.00	0.00
50	100.00	0.00
25	100.00	0.00
12.5	100.00	0.00
6.25	81.35	0.92
3.13	66.42	0.69
1.56	54.31	1.50
0.75	26.34	0.92
0.39	18.1	1.30
0	0.00	0.00
MIC90 (μg/ml)	9.14	
MIC(μg/ml)	12.5	

Table S4 I : Antimicrobial activity of fungus Extract against
Escherichia coli

Sample conc. (µg/ml)	Mean of growth inhibitory%	S.D.
100	100.00	0.00
50	100.00	0.00
25	100.00	0.00
12.5	100.00	0.00
6.25	100.00	0.00
3.13	86.34	0.86
1.56	71.25	1.50
0.75	46.91	2.63
0.39	29.35	1.50
0	0.00	0.00
MIC90 (µg/ml)	3.96	
MIC(µg/ml)	6.25	

Table S4 J: Antimicrobial activity of fungus Extract against
Pseudomonas aeruginosa

Sample conc. (µg/ml)	Mean of growth inhibitory%	S.D.
100	100	0.00
50	100	0.00
25	84.3	1.63
12.5	65.31	1.20
6.25	52.14	2.10
3.13	26.34	0.92
1.56	10.35	1.30
0.75	0.00	0.00
0.39	0.00	0.00
0	0.00	0.00
MIC90 (µg/ml)	34.07	
MIC(µg/ml)	50	

Table S4 K: Antimicrobial activity of fungus Extract against *Aspergillus fumigatus*

Sample conc. (µg/ml)	Mean of growth inhibitory%	S.D.
**100**	**100.00**	**0.00**
**50**	**100.00**	**0.00**
**25**	**86.30**	**1.50**
**12.5**	**71.35**	**0.92**
**6.25**	**54.62**	**1.30**
**3.13**	**29.15**	**1.60**
**1.56**	**16.32**	**2.10**
**0.75**	**0.00**	**0.00**
**0.39**	**0.00**	**0.00**
**0**	**0.00**	**0.00**
**MIC90 (µg/ml)**	**31.75**	
**MIC(µg/ml)**	**50**	

Table S4 L: Antimicrobial activity of fungus Extract against *Candida albicans*

Sample conc. (µg/ml)	Mean of growth inhibitory%	S.D.
**100**	**100.00**	**0.00**
**50**	**100.00**	**0.00**
**25**	**100.00**	**0.00**
**12.5**	**100.00**	**0.00**
**6.25**	**91.35**	**0.63**
**3.13**	**73.52**	**0.92**
**1.56**	**53.47**	**1.50**
**0.75**	**33.28**	**1.30**
**0.39**	**16.34**	**0.63**
**0**	**0.00**	**0.00**
**MIC90 (µg/ml)**	**5.27**	
**MIC(µg/ml)**	**12.5**	

Table S4 M: Antimicrobial activity of Fungus fraction 1

**Sample conc. (µg/ml)**	***Pseudomonas aeruginosa***	***staphylococcus aureus***	***Streptococcus mutans***	***Escherichia coli***	***Candida albicans***
125	100± 0.00	100± 0.00	100± 0.00	100± 0.00	100± 0.00
62.5	100± 0.00	100± 0.00	100± 0.00	100± 0.00	100± 0.00
31.25	83.25± 1.5	100± 0.00	100± 0.00	100± 0.00	100± 0.00
15.63	65.12± 0.92	100± 0.00	90.15± 1.3	100± 0.00	79.35± 1.5
7.81	43.28± 1.3	93.25± 1.5	66.35± 0.63	100± 0.00	51.32± 1.3
3.9	18.85± 0.58	61.25± 2.1	42.15± 1.3	90.35± 0.72	30.25± 0.63
1.95	6.32± 1.2	32.15± 1.3	20.35± 1.2	72.65± 0.63	15.36± 1.3
0.98	0± 0.00	21.32± 0.92	14.63± 1.3	50.32± 1.5	9.32± 0.72
0	0± 0.00	0± 0.00	0± 0.00	0± 0.00	0± 0.00
MIC	62.5	15.63	31.25	7.81	31.25

Table S4 N: Antimicrobial activity of Fungus fraction 2

**Sample conc. (µg/ml)**	***Pseudomonas aeruginosa***	***staphylococcus aureus***	***Streptococcus mutans***	***Escherichia coli***	***Candida albicans***
125	100± 0.00	100± 0.00	100± 0.00	100± 0.00	100± 0.00
62.5	100± 0.00	100± 0.00	100± 0.00	100± 0.00	100± 0.00
31.25	76.32± 0.58	92.13± 0.63	100± 0.00	100± 0.00	100± 0.00
15.63	60.39± 2.1	73.15± 1.5	90.35± 0.58	100± 0.00	83.22± 1.2
7.81	39.29± 1.2	56.32± 1.3	72.14± 2.1	80.96± 1.3	65.38± 0.72
3.9	21.96± 0.63	32.98± 0.92	61.35± 0.63	59.32± 2.1	43.25± 0.63
1.95	10.36± 2.1	19.32± 0.58	49.92± 1.5	26.35± 0.92	32.14± 1.5
0.98	0± 0.00	8.16± 1.5	22.13± 1.2	14.33± 1.3	19.35± 0.58
0	0± 0.00	0± 0.00	0± 0.00	0± 0.00	0± 0.00
MIC	62.5	62.5	31.25	15.63	31.25

Table S4 O: Antimicrobial activity of Fungus fraction 3

**Sample conc. (µg/ml)**	***Pseudomonas aeruginosa***	***staphylococcus aureus***	***Streptococcus mutans***	***Escherichia coli***	***Candida albicans***
125	100± 0.00	100± 0.00	100± 0.00	100± 0.00	100± 0.00
62.5	100± 0.00	100± 0.00	100± 0.00	100± 0.00	100± 0.00
31.25	100± 0.00	100± 0.00	100± 0.00	100± 0.00	100± 0.00
15.63	100± 0.00	100± 0.00	100± 0.00	100± 0.00	100± 0.00
7.81	86.35± 0.58	100± 0.00	100± 0.00	100± 0.00	82.35± 0.58
3.9	52.14± 0.92	91.35± 0.92	100± 0.00	88.65± 1.3	66.92± 1.5
1.95	31.45± 1.6	63.25± 2.1	91.32± 1.3	70.14± 1.5	41.09± 0.63
0.98	15.94± 1.3	41.98± 0.92	68.95± 0.92	58.32± 2.1	22.83± 0.92
0	0± 0.00	0± 0.00	0± 0.00	0± 0.00	0± 0.00
MIC	15.63	7.81	3.9	7.81	15.63

Table S4 P: Antimicrobial activity of Dihydroauroglaucin

**Sample conc. (μg/ml)**	***Pseudomonas aeruginosa***	***staphylococcus aureus***	***Streptococcus mutans***	***Escherichia coli***	***Candida albicans***
125	100± 0.00	100± 0.00	100± 0.00	100± 0.00	100± 0.00
62.5	100± 0.00	100± 0.00	100± 0.00	100± 0.00	100± 0.00
31.25	100± 0.00	100± 0.00	100± 0.00	100± 0.00	100± 0.00
15.63	100± 0.00	100± 0.00	100± 0.00	100± 0.00	100± 0.00
7.81	100± 0.00	100± 0.00	100± 0.00	100± 0.00	100± 0.00
3.9	82.15± 0.92	100± 0.00	100± 0.00	100± 0.00	100± 0.00
1.95	43.21 ± 1.6	92.14± 0.92	100± 0.00	100± 0.00	86.34± 2.1
0.98	21.35± 1.3	69.89± 2.1	93.32± 1.5	86.35± 0.58	56.32± 1.3
0	0± 0.00	0± 0.00	0± 0.00	0± 0.00	0± 0.00
MIC	7.81	3.9	1.95	1.95	3.9

S4 file: Tables of MICs values for antimicrobial activities using microdilution assay
