# Supplementary material for: Bio-Guided Fractionation of Prenylated Benzaldehyde Derivatives as Potent Antimicrobial and Antibiofilm from Ammi majus L. Fruits-Associated Aspergillus amstelodami
Source: Molecules. 2019 Nov 14;24(22):4118. doi: 10.3390/molecules24224118 (PMC6891696; doi:10.3390/molecules24224118)
Supplement: Supplementary file 1 [file molecules-24-04118-s001.zip › S5 fileFigures for Antibiofilm activities against different microorganism strains.pdf]

II- Antibiofilm activity

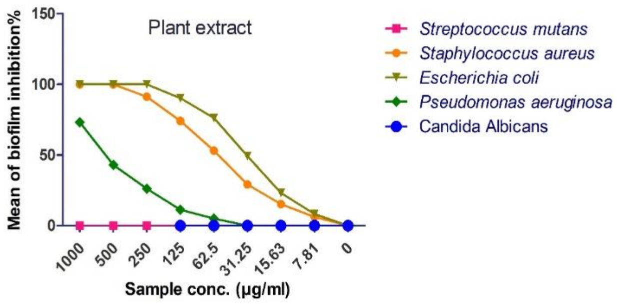

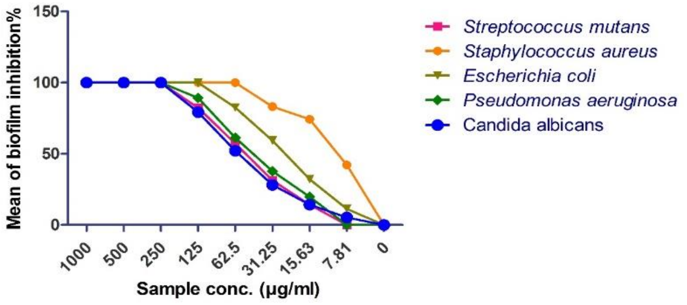

Antibiofilm activity of the plant extract

Antibiofilm activity of the fungal extract

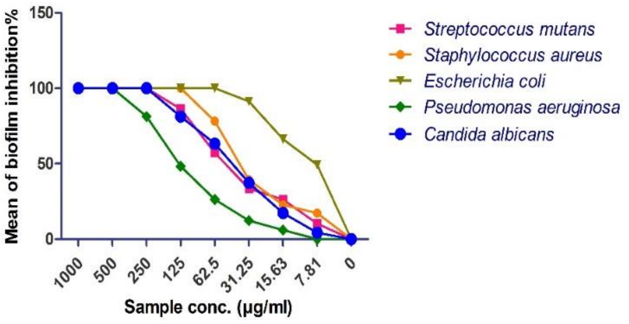

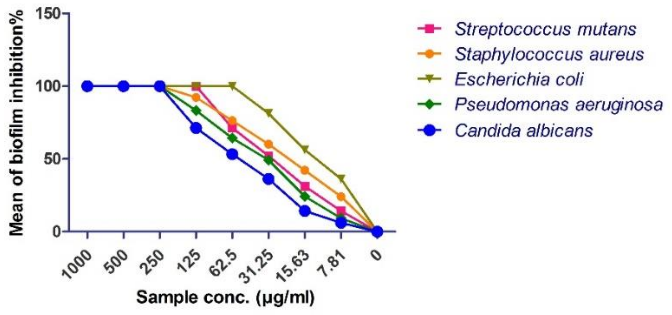

Antibiofilm activity of fraction 1

Antibiofilm activity of fraction 2

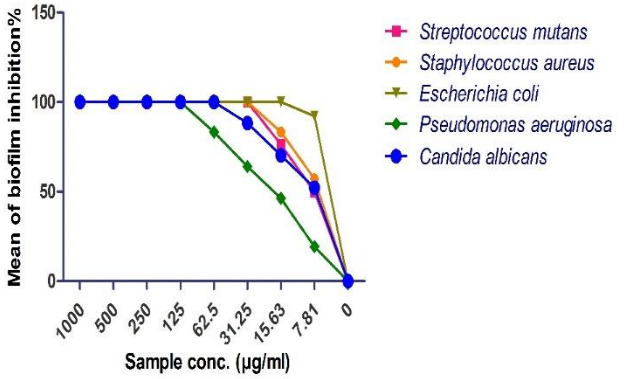

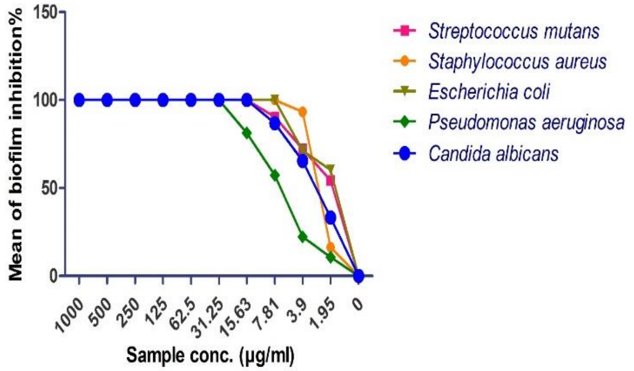

Antibiofilm activity of fraction 3

Antibiofilm activity of Dihydroauroglauc
