# Supplementary figures and images for: Bio-Guided Fractionation of Prenylated Benzaldehyde Derivatives as Potent Antimicrobial and Antibiofilm from Ammi majus L. Fruits-Associated Aspergillus amstelodami
Source: Molecules. 2019 Nov 14;24(22):4118. doi: 10.3390/molecules24224118 (PMC6891696; doi:10.3390/molecules24224118)

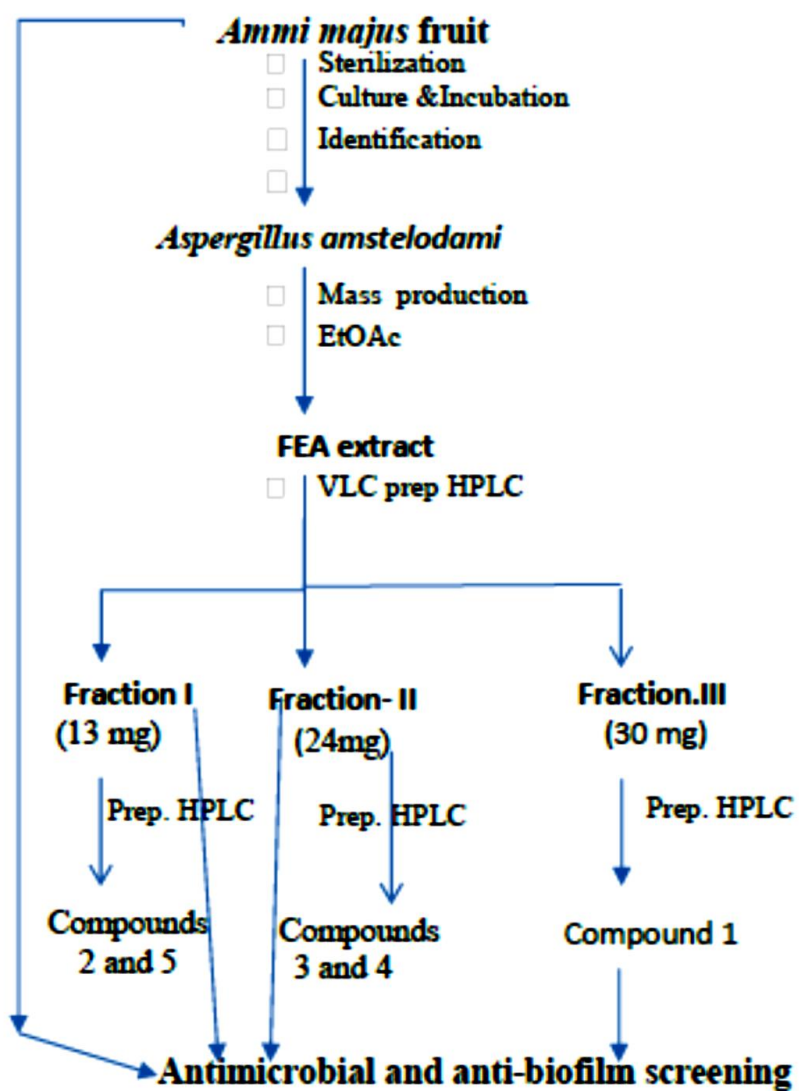

S7 file: Schematic Fractionation protocol

Supplement: Supplementary file 1 [file molecules-24-04118-s001.zip › S7 file Schematic Fractionation protocol.pdf]
